# A phase I, randomized study to evaluate the safety, tolerability, and pharmacokinetics of mefunidone in healthy subjects

**DOI:** 10.3389/fphar.2024.1414066

**Published:** 2024-06-12

**Authors:** Mai Han, Bishan Huo, Gaoyun Hu, Xin Zhang, Gang Cui, Wei Wu, Na Mi, Shixi Zhang, Jiangli Jin, Xing Lu, Bidong Wu, Chunyan Xiao, Jing Wang, Zheng Bian, Jintong Li

**Affiliations:** ^1^ Drug Clinical Trial Research Center, China-Japan Friendship Hospital, Beijing, China; ^2^ Guangzhou Nanxin Pharma Co., Ltd., Guangzhou, China; ^3^ Xiangya School of Pharmaceutical Science, Central South University, Changsha, China

**Keywords:** mefunidone, pharmacokinetics, first-in-human, safety, food effect

## Abstract

**Background:**

Mefunidone is a novel synthetic compound and is better when compared to pirfenidone for the anti-fibrotic treatment of renal fibrosis in end-stage renal disease. We conducted this first-in-human, phase I clinical trial to determine the safety, tolerability, and pharmacokinetic (PK) (including food effect) profiles of mefunidone administered orally as single and multiple ascending doses in healthy subjects.

**Methods:**

Part A assessed single ascending doses of mefunidone from 25 mg to 800 mg or placebo once daily in the fasting state. Part A also assessed the effect of food on tolerability and PK in the 100 mg cohort. Part B consisted of three treatment groups who received 100 mg, 200 mg, or 400 mg of mefunidone or placebo twice daily (BID, *bis in die*) on days 1–6 and once in the morning on day 7.

**Results:**

Single oral doses of mefunidone up to 800 mg and multiple doses of mefunidone up to 400 mg BID were all well-tolerated. Mefunidone behaved with ideal dose proportionality within the single-dose range of 50 mg–600 mg and the multiple-dose range of 100 mg BID to 400 mg BID by day 7. High-fat fed conditions led to a delay in T_max_ by approximately 1 h and a slight reduction of approximately 20% in C_max_ compared to that in fasting conditions, but it did not significantly affect systemic exposure.

**Conclusion:**

Mefunidone exhibited favorable pharmacokinetics and safety profiles. The present study informed and supported further developmental clinical studies of mefunidone.

**Clinical Trial Registration:**

clinicaltrials.gov, identifier CXHL1900206

## 1 Introduction

Diabetic kidney disease (DKD), a kind of microvascular complication, is the primary cause of end-stage renal disease (ESRD) worldwide (Forbes and Cooper, 2013; Liu et al., 2019). Type 1 diabetes mellitus (T1D) and type 2 diabetes mellitus (T2D) are the most common causes of DKD. The incidence of DKD complicated by T1D and T2D is approximately 50% and 30%–40%, respectively (Tuomilehto et al., 1998; Retnakaran et al., 2006; Gheith et al., 2016). Renal tubulointerstitial fibrosis is the final common pathway of this disease (Zhang et al., 2012). It is characterized by the accumulation of the extracellular matrix (ECM) in the renal interstitium. Limited therapeutic options for renal tubulointerstitial fibrosis are available (Boor et al., 2010). Drugs for treating patients with renal fibrosis have not been approved yet (Huang et al., 2023).

Pirfenidone is an anti-fibrotic that reduces fibroblast proliferation and collagen synthesis in animal models and is associated with reductions in various oxidative, inflammatory, and fibrogenic biomarkers (Schaefer et al., 2011). It is recognized as the most promising anti-fibrotic drug in clinical application (Regulatory Watch, 2008) and has been approved for the treatment of idiopathic pulmonary fibrosis (IPF) (Xaubet et al., 2014). Furthermore, pirfenidone has been proven to be effective in patients with focal segmental glomerulosclerosis (Cho et al., 2007) or diabetic nephropathy (Sharma et al., 2011). However, results of previous research studies have suggested that pirfenidone has inadequate pharmacological activity and a high first‐pass effect due to the induction of hepatic enzymes. In order to increase the *in vivo* bioavailability of pirfenidone, the recommended daily dosage is high, approximately 1,200 mg/d, with a relatively short dosing interval (King et al., 2014; Karimishah and Chowdhury, 2015; Noble et al., 2016). The high dosage is also related to limitations in tolerability, with increased rates of photosensitivity, fatigue, stomach discomfort, and anorexia compared to placebo.

**FIGURE 1:**
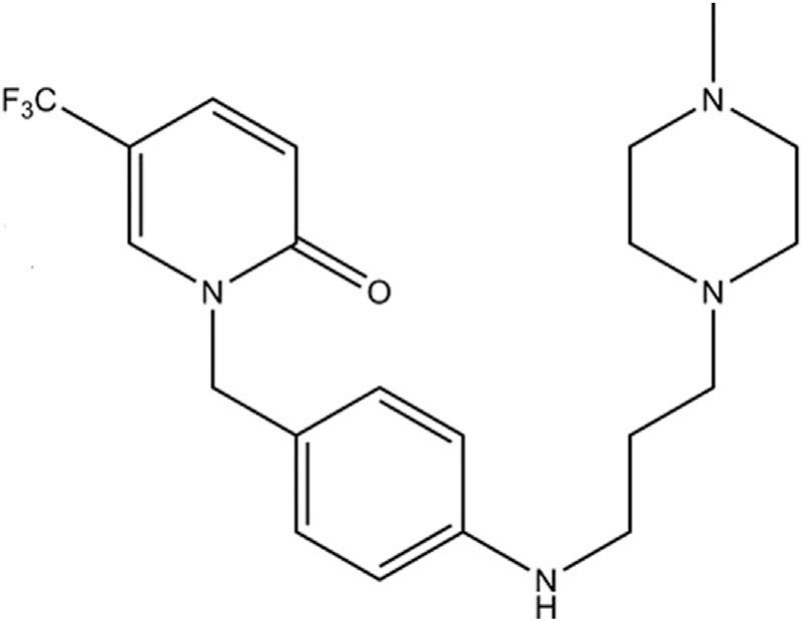
Chemical structure of mefunidone.

Mefunidone (MFD) [1-(4-((3-(4-methylpiperazin-1-yl)propyl)amino)benzyl)-5-(trifluoromethylpyridin-2(1H)-one) is a novel synthetic compound and is preferred to pirfenidone (Figure 1). In contrast with pirfenidone, whose main is 5-carboxy-pirfenidone, MFD has the structure 5-trifluoromethyl-2-pyridone, where hydrogen atoms on the methyl are substituted by fluorine atoms, preventing the CH3 group from forming carboxyl groups. In addition, the hydrogen atom in the para-position of the benzene ring of pirfenidone is substituted by the nitrogen atom in the structure of 1-(3-aminopropyl)-4-methylpiperazine (Luo et al., 2021). These structural modifications are considered to bring about several advantages that make mefunidone superior to pirfenidone. 1) Mefunidone has better metabolic stability (Han et al., 2019; Luo et al., 2021). Preclinical investigations revealed that mefunidone does not induce or inhibit metabolic enzymes, i.e., CYP3A4, CYP2C9, and CYP2C8 (Han et al., 2019; Luo et al., 2021). In this way, mefunidone avoids the drawback of the high first‐pass effect of pirfenidone due to the induction of hepatic enzymes (Han et al., 2019; Luo et al., 2021). In addition, results revealed satisfactory absorption of mefunidone *in vivo*, as well as improved solubility, oral bioavailability, and longer half-life compared with pirfenidone (Wu, 2013; Yang, 2014; Liu et al., 2015; Han et al., 2019; Jiang et al., 2021). 2) In preclinical studies, there were limitations in the tolerability of pirfenidone, such as increased rates of photosensitivity, fatigue, stomach discomfort, and anorexia compared to placebo (Raghu et al., 2015). In contrast, mefunidone exhibited lower toxicity (Han et al., 2021; Jiang et al., 2021). 3) Mefunidone showed better anti-fibrosis and anti-inflammatory effects in unilateral urethral obstruction (UUO) animal models and several cell lines (Liu et al., 2015; Chen et al., 2018). The anti-fibrotic effect of mefunidone was estimated to be 20-fold stronger than that of pirfenidone (Liu et al., 2015). These findings suggest that mefunidone is a promising candidate molecule for the anti-fibrotic treatment of CKD.

The primary objective of this first-in-human study was to determine the safety and tolerability of mefunidone administered orally in single and multiple ascending doses to healthy subjects. The secondary objectives were to determine the pharmacokinetic profiles following the single and multiple oral doses of mefunidone and to evaluate the effects of food on the tolerability and PK of mefunidone after the administration of a single oral dose in healthy subjects.

## 2 Materials and methods

### 2.1 Study design

This randomized, double-blinded, placebo-controlled, ascending-dose trial (100 mg: CXHL1900206; 25 mg: CXHB1900060), sponsored by Nanxin Corporation (Guangdong, China) and Central South University (Hunan, China), was conducted at a single clinical research site in the China–Japan Friendship Hospital (Beijing, China) to study the safety, tolerability, and PK profiles of a single dose (Part A) and multiple doses (Part B) of mefunidone in healthy subjects. In Part A, the effect of food on the PK of a single dose was also assessed in the same healthy subjects with a randomized sequence of administration (fasted–fed or fed–fasted) across two different treatment periods.

Each subject receiving mefunidone or the placebo was determined by the statistician using SAS 9.4 to generate a random allocation table. In Part A, Cohorts A1 and A7 included a sentinel cohort of two subjects each, randomly assigned to receive either mefunidone or placebo (1 mefunidone: 1 placebo). All other subjects were randomized to mefunidone *versus* placebo using the following allocation: the remainders of A1 and A7, 3:1; A2, A4, and A5; 4:1, and A3, 6:1. Since the placebo was different in appearance from the mefunidone tablets, dosing was performed by two authorized, independent, unblinded investigators, and all subjects, other investigators, and staff remained blinded to the treatment randomization code throughout the study to preserve blinding.

In Part A, the single ascending-dose (SAD) phase, 64 subjects were studied in seven dosing cohorts (A1 to A7). The A1 and A7 cohorts had six subjects each (two mefunidone: two placebo). The A2, A4, and A5 cohorts had 10 subjects each (eight mefunidone: two placebo), the A6 cohort had eight subjects (six mefunidone: two placebo), and the A3 cohort had 14 subjects (12 mefunidone: two placebo). Four subjects in the A1 and A7 cohorts (two in each cohort, one mefunidone: one placebo) served as sentinel subjects dosed at least 2 weeks ahead of the remainder of the cohort. During Period 1, A1–A7 cohorts received single, ascending doses of mefunidone or placebo at the following levels: A1, 25 mg; A2, 50 mg; A3, 100 mg; A4, 200 mg; A5, 400 mg; A6, 600 mg; and A7, 800 mg. Dosing occurred on day 1 in the morning after overnight fasting of at least 10 h (except for subjects in cohort A3 that were assigned to the fed–fasted sequence; see below). Subjects in cohort A3 participated in an additional treatment period (Period 2). The 14 subjects in this cohort were assigned to the fasted–fed or fed–fasted sequence according to the randomization schedule (seven subjects for each sequence), and they received another dose of mefunidone 100 mg or placebo on day 8. Subjects assigned to the fed conditions consumed a standard high-fat, high-calorie breakfast (approximately 800–1,000 calories in total, with fat accounting for approximately 50 percent of total energy) before dosing. A wash-out period of 7 days separated the two treatment periods in the A3 cohort. All subjects were confined to the clinical research unit from day 1 to day 4 (72 h post-dose) of each treatment period. The subjects with unresolved AEs returned for follow-up visits after their discharge.

**FIGURE 2:**
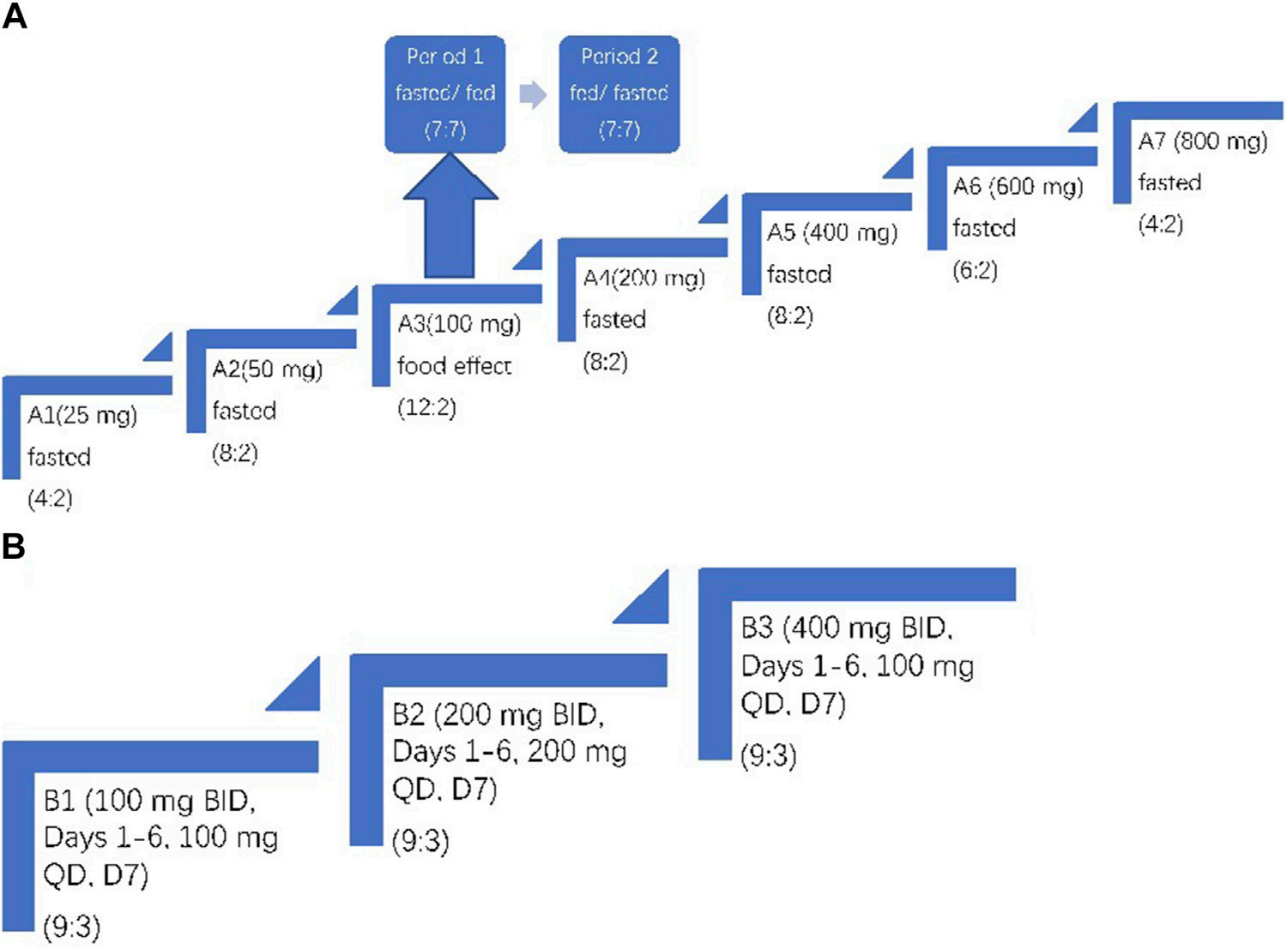
Trial design. **(A)** Part A: single ascending-dose study with treatment administered on day 1. In Period 1 and Period 2 of A3, subjects were fasted or fed before mefunidone administration. **(B)** Part B: multiple ascending-dose study with treatment administered on day 1 through day 7. The treatment allocation for each cohort is shown as (active: placebo). For A3, the same group of subjects was dosed over two treatment periods.

Part B, the multiple ascending-dose (MAD) phase, started after a review of safety, tolerability, and PK data from Part A. A total of 36 subjects were studied in three cohorts (B1, 100 mg; B2, 200 mg; and B3, 400 mg). Each cohort had 12 subjects (nine mefunidone: three placebo). All subjects were orally administered with mefunidone or placebo twice daily (BID, *bis in die*) on days 1–6 and once in the morning on day 7. Dosing on day 1 morning and D7 morning was performed after overnight fasting of at least 10 h. The other doses were administered after a standard meal (Figure 2).

### 2.2 Ethics

The study protocol and informed consent form were approved by the Clinical Research Ethics Committee of the China–Japan Friendship Hospital. The study was conducted in accordance with the principles of Good Clinical Practice and the Declaration of Helsinki. Written informed consent had been obtained from all the subjects before enrollment.

### 2.3 Subjects

Subjects were male or female healthy volunteers, 18–65 years old, with a minimal body weight of 50.0 kg for males and 45.0 kg for females. The body mass index (BMI) required was between 19.0 and 28.0 kg/m^2^. The key inclusion criteria required subjects to be assessed as healthy by a review of their medical history, physical examination, vital sign measurements, 12-lead electrocardiogram (ECG), and clinical laboratory evaluations. Subjects were excluded if they had any known history of allergy, prior or present evidence of any organopathy (heart disease, liver or kidney disease, etc.), drug or alcohol abuse, or infectious diseases, nor could they have any use, or intent to use, of medications, including prescription, over-the-counter, herbal preparations, or vitamin/mineral supplementation, other than the study medications, from 14 days before the first study dose through the completion of the follow-up visits or participation in another clinical trial within 3 months of the other investigative drug before the first dose of the study drug on day 1.

### 2.4 Dose selection

In accordance with the FDA (Food and Drug Administration) and NMPA (National Medical Products Administration) guidance recommendations (FDA, 2005), the maximum recommended starting dose (MRSD) was determined based on the no-observed-adverse-effect level (NOAEL) in a 4-week Good Laboratory Practice toxicity study. Conservatively, using the more sensitive toxicological species (rat), the NOAEL was 200 mg/kg/day (unpublished data), which corresponds to a human equivalent dose (HED) of 36 mg/kg/day and an MRSD of 3.6 mg/kg/day (HED/10). In addition, the NOAEL in the reproductive study of rats was 50 mg/kg/day, which corresponds to an HED of 9 mg/kg/day and an MRSD of 0.9 mg/kg/day (HED/10). Based on these results, we selected the starting single dose of 25 mg (0.42 mg/kg for a 60-kg subject), which had an 85-fold safety margin compared with the HED extrapolated from the rat NOAEL and a 21-fold safety margin compared with the HED for reproductive safety in rats.

The 4-week Good Laboratory Practice chronic toxicology studies also revealed that the lowest-observable adverse effect dose was 400 mg/kg/day for rodents (rat) and 150 mg/kg for non-rodents (Cynomolgus monkey), which corresponds to a HED of 60–71.4 mg/kg/day. The proposed highest single dose of 800 mg (13.3 mg/kg for a 60-kg subject) had a 2.7-fold safety margin compared with the HED.

### 2.5 Safety assessments

To establish the safety profile of mefunidone, this trial monitored treatment-emergent adverse events (TEAEs) coded according to the Medical Dictionary of Regulatory Activities (MedDRA, Version 18.0), physical examinations, vital sign measurements, clinical laboratory evaluations, and 12-lead ECGs. Doses in Part A of the study were escalated until the maximum tolerated dose was achieved or any of the stopping criteria were met. The site investigator and the sponsor’s site monitoring committee (SMC), consisting of experts in clinical science, biostatistics, clinical pharmacology, and drug safety, escalated doses after a satisfactory review of the safety, tolerability, and PK data (at least 2 weeks post-dose). Sequential dose increments did not exceed the previous dose by more than a factor of 2. Dose escalation would be stopped if the following events occurred:

(1) More than half of the subjects had TEAEs with a severity of grade 2.
(2) More than a quarter of the subjects had TEAEs with a severity of grade 3.
(3) Two similar (by preferred term [PT]) treatment-related grade 3 AEs.
(4) One treatment-related SAE.
(5) PK analysis revealing the saturation of systemic exposure to mefunidone, i.e., the AUC did not increase along with dose escalation

### 2.6 Pharmacokinetic assessments

Blood samples were collected at different time points and then analyzed to determine the concentration of mefunidone. In Part A, blood samples were collected prior to dosing and at various time points up to 72 h post-dose. In Part B, blood samples were collected prior to dosing on days 1, 5–6, and 7, up to 12 h post-dose on D1, and up to 72 h post-dose on D7. Mefunidone plasma concentrations were measured by a liquid chromatography–tandem mass spectrometry (LC-MS/MS) method that was validated in human plasma.

### 2.7 Pharmacokinetic analysis

The PK parameters were calculated where possible from plasma concentrations of mefunidone at actual sampling times by using non-compartmental analysis with Phoenix WinNonlin 8.2. The following PK parameters were determined: area under the plasma concentration–time curve from time 0 to the time of last quantifiable concentration (AUC_0-t_); area under the concentration–time curve from time 0 extrapolated to infinity (AUC_0-inf_), maximum observed plasma concentration (C_max_); time of maximum observed plasma concentration (T_max_); apparent plasma terminal elimination half-life (T_1/2_); apparent total plasma clearance (CL/F); and apparent volume of distribution during the terminal elimination phase (Vz/F). Data for C_max_ and AUC_0-t_ of D1 and steady state were compared in order to determine the accumulation ratio of mefunidone (Rac_C_max_ and Rac_AUC).

### 2.8 Statistical methods

The safety population consisted of all subjects who received any amount of the study drug or placebo and had at least one post-dose safety assessment. For Parts A and B, summaries of TEAEs for the subjects assigned to placebo were pooled across all dose cohorts and treatment periods. The PK population consisted of all subjects who received any amount of the study drug and had at least one evaluable PK sample. The bioequivalence set (BES) consisted of all A3 subjects who received both doses in the food effect study and had evaluable PK samples in two periods. Data analysis was performed using SAS^®^ Version 9.4 (SAS Institute Inc., Cary, NC). The baseline was defined as the last pre-dose measurement.

Dose proportionality was assessed using a power model on natural log-transformed C_max_ and AUC:

$$\ln\left(y\right)=\alpha +\beta \times \ln\left(\text{Dose}\right),$$

where y represents C_max_, AUC_0-t,_ and AUC_0-inf_, respectively; α represents the intercept term; and β represents the slope. The judgment interval is 1+ln (θ L)/ln r)∼1+ln (θ H)/ln r), where θ H = 1.25, θ L = 0.8, and r is the ratio of the highest dose to the lowest dose.

Considering the small sample size, dose-normalization was also used as a compensative method to assess dose proportionality.

To assess the effects of food on exposure and PK parameters (AUC_0-t_, AUC_0-∞_, and C_max_), the parameters under the fasted condition served as the reference treatment, and the parameters under the fed condition served as the test treatment. The 90 percent CI for the ratio of population geometric means between the test and reference products was provided for AUC_0-inf_, AUC_0-t_, and C_max_.

## 3 Results

### 3.1 Subject dispositions and demographics

The study began in September 2020, at the time of the first informed consent, and ended in April 2022, the date of LPLO (last patient/subject last out). A total of 100 subjects (64 for Part A and 36 for Part B) entered the study and were randomized. The study population consisted of 58 male and 42 female subjects. In Part A, of the 64 subjects enrolled (36 men and 28 women; 50 mefunidone and 14 placebo), five were of ethnicity other than Han Chinese (three Man Chinese, one Zhuang Chinese, and one Hui Chinese). In Part B, of the 36 subjects enrolled (22 male and 14 female; 27 mefunidone and 9 placebo), three were of ethnicity other than Han Chinese (one Man, one Mongolian, and one Bouyei Chinese). The height, weight, and BMI were similar across cohorts (Table 1).

**TABLE 1:** Subject demographics in Part A.

Part A demographic	Pooled placebo (N = 14)	Mefunidone							Active total (N = 50)	All subjects (N = 64)
		A1 (25 mg) N = 4	A2 (50 mg) N = 8	A3 (100 mg) N = 12	A4 (200 mg) N = 8	A5 (400 mg) N = 8	A6 (600 mg) N = 6	A7 (800 mg) N = 4		
Age (years), mean (SD)	31.9 (5.9)	30.0 (9.6)	28.4 (6.0)	31.0 (5.7)	32.0 (6.4)	25.4 (4.5)	38.3 (5.7)	31.3 (2.8)	30.7 (6.5)	30.9 (6.4)
Weight (kg), mean (SD)	69.6 (10.7)	65.8 (5.3)	62.5 (8.8)	63.6 (6.9)	66.0 (10.4)	69.1 (11.7)	61.2 (6.7)	55.8 (6.6)	63.9 (8.7)	65.2 (9.5)
Height (cm)	168.7 (6.5)	167.8 (5.7)	164.9 (4.6)	163.8 (6.2)	165.3 (9.3)	173.3 (8.3)	160.8 (5.2)	159.3 (6.1)	165.3 (7.5)	166.1 (7.4)
BMI (kg/m^2^), mean (SD)	24.3 (2.6)	23.4 (2.3)	22.9 (2.1)	23.7 (2.1)	24.0 (1.7)	22.9 (2.7)	23.6 (2.1)	22.0 (2.1)	23.3 (2.1)	23.5 (2.2)
Sex, male, n (%)	10 (71.4)	2 (50.0)	5 (62.5)	5 (41.7)	5 (62.5)	7 (87.5)	2 (33.3)	0	26 (52.0)	36 (56.3)
Ethnicity, non-Han Chinese, n (%)	0	1 (25.0)	0	2 (16.7)	0	1 (12.5)	1 (16.7)	0	5 (10.0)	5 (7.8)

Part B demographic	Pooled placebo (n = 9)	Mefunidone			Active total (n = 27)	All subjects (n = 36)
		B1 (100 mg) (n = 9)	B2 (200 mg) (n = 9)	B3 (400 mg) (n = 9)		
Age (years), mean (SD)	28.0 (5.8)	33.3 (5.3)	29.6 (4.9)	30.7 (4.8)	31.2 (5.0)	30.4 (5.4)
Weight (kg), mean (SD)	62.1 (6.0)	64.9 (11.2)	67.1 (6.6)	62.7 (6.7)	64.9 (8.2)	64.2 (7.8)
Height (cm)	166.6 (6.8)	166.9 (10.2)	167.1 (8.0)	165.6 (6.1)	166.5 (7.8)	166.6 (7.6)
BMI (kg/m^2^), mean (SD)	22.1 (1.5)	23.2 (2.8)	23.9 (1.5)	22.6 (1.9)	23.2 (2.1)	23.0 (2.0)
Sex, male, n (%)	5 (55.6)	6 (66.7)	6 (66.7)	5 (55.6)	17 (63.0)	22 (61.1)
Ethnicity, non-Han Chinese, n (%)	1 (11.1)	0	0	2 (22.2)	2 (7.4)	3 (8.3)

One subject in Part A and two subjects in Part B withdrew due to TEAEs (see below), and the other subjects received their full dose of mefunidone or placebo at the appropriate number of either 25- or 100-mg tablets. Except for one subject who was lost to follow-up, all the other subjects completed the study.

One subject in the A7 cohort (ID: 705) vomited at 74 min after taking mefunidone. Since this event happened within twice the median T_max_ (1.25 h) for the A7 cohort, the subject’s data were excluded from the Pharmacokinetic Analysis Concentration Set (PKCS) and the Pharmacokinetic Analysis Parameter Set (PKPS). In the food effect study, one subject withdrew due to adverse events (ID: 308), and her data were excluded from the bioequivalence analysis set (BES).

### 3.2 Safety

In Part A (SAD and food effect), one subject withdrew due to a TEAE (308 in the A3 cohort; see below). No deaths, SAEs, dose-limiting adverse events, or pregnancies occurred during the study. In Part A (Period 1), single oral doses of mefunidone ranging from 25 to 800 mg were administered in the fasted state. Overall, 23 mefunidone-treated subjects (46.0%, fasted state) and four placebo-treated subjects (28.6%, across all periods) experienced TEAEs (Supplementary Table S1). All Part A TEAEs observed in subjects in the fasted state were mild and were considered related to treatment. Except for one loss to follow-up (308 in the A3 100 mg cohort), all TEAEs were resolved by the end of the study without further intervention. The most frequent AEs were dizziness and nausea, which were experienced by eight (16%) and five (10%) mefunidone-treated subjects, respectively.

When food was introduced at the 100 mg mefunidone dose level (Period 2), the incidence of TEAEs was similar. There were six (50.0%) subjects in the fasted state and six (54.5%) subjects in the high-fat-fed state who experienced TEAEs (Supplementary Table S2). No placebo-treated subjects experienced TEAEs in this group. At the end of Period 1, one subject in the fasted state had mild anemia (hemoglobin 10.6 g/dL). The investigators terminated her participation for ethical concerns and planned to follow up on the outcome of the anemia. However, the subject disconnected from the investigators and was lost to follow-up.

In Part B, 22 mefunidone-treated subjects (81.5%) and eight placebo-treated subjects (88.9%) experienced TEAEs (Supplementary Table S3). One mefunidone-treated subject (11.1%) in the B2 cohort and one mefunidone-treated subject (11.1%) in the B3 cohort had six TEAEs of moderate degree (hypertriglyceridemia, alanine aminotransferase increase, and dizziness in the B2 cohort and tonsillitis, hyponatremia, and alanine aminotransferase increase in the B3 cohort). Two subjects (22.2%) in the B3 cohort had TEAEs that led to withdrawal (maculo-papular rash and tonsillitis). All the other TEAEs were mild. All Part B TEAEs were considered related to treatment, and they had resolved by the end of the study.

### 3.3 Part A: single ascending-dose pharmacokinetics

#### 3.3.1 Part A: period 1 PK parameters

**FIGURE 3:**
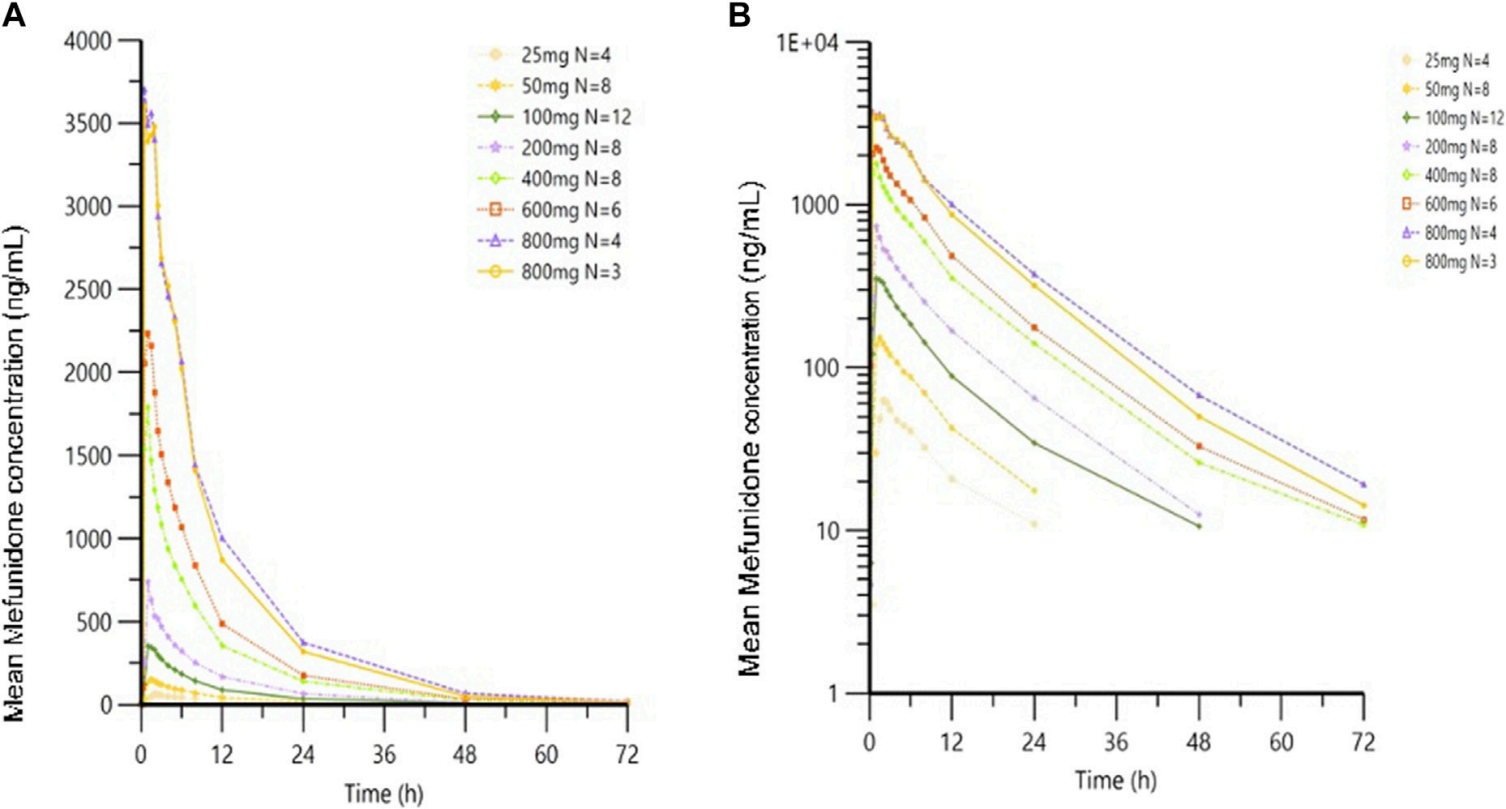
Mean plasma concentrations of mefunidone in the fasted state following single-dose administration. Data are plotted on **(A)** linear and **(B)** semi-logarithmic scales.

Following oral administration of single doses of mefunidone up to 800 mg in the fasted state, mefunidone was readily absorbed and reached plasma C_max_ with median T_max_ values ranging from 0.8 to 2.0 h, followed by a one-exponential decline, and ended with parallel lines for the elimination phase across all dose groups; the mean T_1/2_ ranged from 7.5 to 9.9 h between 25 mg and 800 mg doses (Figure 3; Table 2).

**TABLE 2:** Pharmacokinetic parameters of mefunidone by treatment group in Part A (period 1; single doses administered in the fasting state).

Parameter	A1 (25 mg) N = 4	A2 (50 mg) N = 8	A3 (100 mg) N = 12	A4 (200 mg) N = 8	A5 (400 mg) N = 8	A6 (600 mg) N = 6	A7 (800 mg) N = 3
AUC_0-t_ (h*ng/mL)	513.9 (26.6)	1,328.9 (15.2)	2,811.1 (24.1)	5,985.3 (15.2)	14,089.3 (13.4)	19,318.6 (13.1)	34,331.3 (11.9)
AUC_0-∞_ (h*ng/mL)	789.0 (3.2)	1,544.7 (13.7)	3,184.6 (22.4)	6,162.3 (14.8)	14,330.8 (13.0)	19,566.4 (12.6)	34,532.6 (12.1)
%AUC_extrap_ ^a^ (%)	19.5 (0.6)	11.9 (2.7)	11.7 (3.3)	2.9 (0.6)	1.7 (8.0)	1.3 (0.7)	0.6 (0.2)
C_max_ (ng/mL)	64.5 (10.0)	167.4 (26.9)	351.6 (27.1)	733.2 (20.8)	1,865.8 (16.2)	2,644.8 (30.2)	4,075.5 (28.0)
t_max_ ^b^ (h)	2.0 (1.5–2.5)	1.0 (1.0–3.0)	1.8 (1.0–4.0)	1.0 (1.0–1.5)	1.0 (0.5–1.0)	0.8 (0.5–1.5)	2.0 (0.5–2.5)
t_1/2_ (h)	9.7 (6.9)	7.5 (12.7)	7.9 (10.6)	9.7 (7.2)	9.8 (14.8)	9.9 (12.0)	9.9 (7.7)
CL/F (mL/h)	31,685.2 (3.2)	32,367.9 (13.7)	31,401.5 (22.4)	32,455.3 (14.8)	27,911.9 (13.0)	30,664.9 (12.6)	23,166.5 (12.1)
V_z_/F (L)	444.4 (3.7)	347.9 (21.8)	358.5 (21.2)	452.6 (20.2)	394.2 (11.2)	438.8 (16.5)	331.3 (4.8)

AUC_0-t_, the area under the concentration–time curve (AUC) from time 0 to the last quantifiable concentration; AUC_0-∞_, AUC extrapolated to infinity.

%AUC_extrap_, percentage of AUC that is due to extrapolation from the last measurable concentration to infinity; C_max_, maximum observed plasma concentration.

CL/F, apparent total plasma clearance; CV%, coefficient of variation; n, number of subjects; t_max_, time to maximum concentration.

t_1/2_, apparent terminal elimination half-life; V_z_/F, apparent volume of distribution during the terminal elimination phase.

Data are geometric means (geometric CV%), unless otherwise indicated.

^a^ Arithmetic mean (arithmetic standard deviation).

^b^ Median (range).

Over the dose range of 25 mg–800 mg, systemic exposure to mefunidone (based on AUC) was not completely linearly related to increases in the administered doses. When the dose range was restricted to 50 mg–600 mg, a proportional increase in C_max_ and AUC could be observed. The slopes for C_max_, AUC_0-t_, and AUC_0-inf_ were 1.136 (90% CI 1.064–1.208), 1.104 (90% CI 1.052–1.157), and 1.040 (90% CI 0.989–1.092), respectively. Better proportionality was observed in the dose range of 100 mg–400 mg, with slopes of 1.195 (90% CI 1.070–1.321), 1.158 (90% CI 1.054–1.263), and 1.077 (90% CI 0.976–1.178) for C_max_, AUC_0-t_, and AUC_0-inf_, respectively (Table 3). Apparent total plasma clearance, CL/F, was similar in the 25 mg–600 mg dose groups, with geometric mean values of 465.2–540.9 mL/min, but decreased in the 800-mg dose group (386.1 mL/min). Apparent volume of distribution, V_z_/F, was similar across dose groups, ranging from 331.3 L to 452.6 L. Inter-subject variability in AUC_0-t_, AUC _0-∞_, and C_max_ was generally low to moderate across the 25 mg–800 mg dose range, with geometric %CV of 11.9%–26.6% for AUC_0-t_, 3.2%–22.4% for AUC _0-∞_, and 10.0%–30.2% for C_max_ (Table 2).

**TABLE 3:** Dose proportionality assessment of mefunidone in the SAD and MAD studies (PKPS).

Parameters	Estimated values (β)	90% CIs
Mefunidone SAD study (25 mg–800 mg)		
C_max_	1.162	1.108, 1.215
AUC_0-t_	1.147	1.105, 1.190
AUC_0-inf_	1.059	1.017, 1.101
Mefunidone SAD study (50 mg–600 mg)		
C_max_	1.136	1.064, 1.208
AUC_0-t_	1.104	1.052, 1.157
AUC_0-inf_	1.040	0.989, 1.092
Mefunidone SAD study (100 mg–400 mg)		
C_max_	1.195	1.070, 1.321
AUC_0-t_	1.158	1.054, 1.263
AUC_0-inf_	1.077	0.976, 1.178
Mefunidone MAD study (100 mg BID- 400 mg BID)		
C_max,ss_	0.966	0.853, 1.080
AUC_0-t,ss_	0.988	0.871, 1.104
AUC_0-∞,ss_	0.983	0.864, 1.101

Algorism: Ln (PK parameter) = α + β × Ln (dose).

AUC_ss_, area under the plasma concentration–time curve during a dosing interval at steady state; CI, confidence interval.

C_max ss_, maximum concentration in plasma at steady state; PKPS, pharmacokinetic parameter set.

#### 3.3.2 Part A: food effect

Administration of mefunidone to subjects under high-fat-fed conditions (Period 2) resulted in a slight decrease in C_max_ and AUC (AUC_0-t_ and AUC_0-∞_) compared with those in fasted conditions (Table 4). The ratio of geometric least squares (LS) means (expressed as a percent) between the high-fat fed state and the fasting state at 100 mg mefunidone were 79.3% (90% CI, 70.2–89.6), 90.38% (90% CI, 85.43–95.62), and 92.6% (90% CI, 88.6–96.7) for C_max_, AUC_0-t_, and AUC_0-∞_, respectively (Table 4). A delay in T_max_ of 1.0 h (median T_max_, 2.0 h) was observed in the high-fat fed group compared to the fasted states (median T_max_, 1.0 h) (Table 5).

**TABLE 4:** Effect of high-fat fed states on 100 mg mefunidone pharmacokinetics.

	LS means	LS means			
Parameters	100 mg mefunidone high-fat fed (test)	100 mg mefunidone fasted (reference)	Test/reference (%)	90% CI (%)	CV (%)
AUC_0-t_ (h*ng/mL)	2606.9	2884.4	90.4	85.4, 95.6	7.2
AUC_0-∞_	3000.0	3240.0	92.6	88.6, 96.7	5.6
C_max_ (ng/mL)	302.0	381.0	79.3	70.2, 89.6	15.7

**TABLE 5:** Pharmacokinetic parameters of 100 mg mefunidone by treatment condition (Part A, single doses in the fasted or high-fat fed state).

	Mefunidone	
Parameter	100 mg (A3) fasted (N = 12)	100 mg (A3) high-fat-fed (N = 11)
AUC_0-t_ (h*ng/mL)	2811.1 (24.1)	2631.1 (25.3)
AUC_0-∞_ (h*ng/mL)	3184.6 (22.4)	3027.3 (23.5)
C_max_ (ng/mL)	351.6 (27.1)	305.6 (27.9)
t_max_ ^a^ (h)	1.8 (1.0-4.0)	2.0 (1.5-6.0)
t_1/2_ (h)	7.9 (10.6)	8.22 (14.0)

AUC_0-t_, area under the concentration–time curve (AUC) from time 0 to the last quantifiable concentration.

AUC_0-∞_, AUC extrapolated to infinity; C_max_, maximum observed plasma concentration; t_max_, time to maximum concentration.

CV%, coefficient of variation; t_1/2_, apparent terminal elimination half-life.

Data are geometric means (geometric CV%), unless otherwise indicated.

^a^ Median (range).

### 3.4 Part B: multiple ascending-dose pharmacokinetics

**FIGURE 4:**
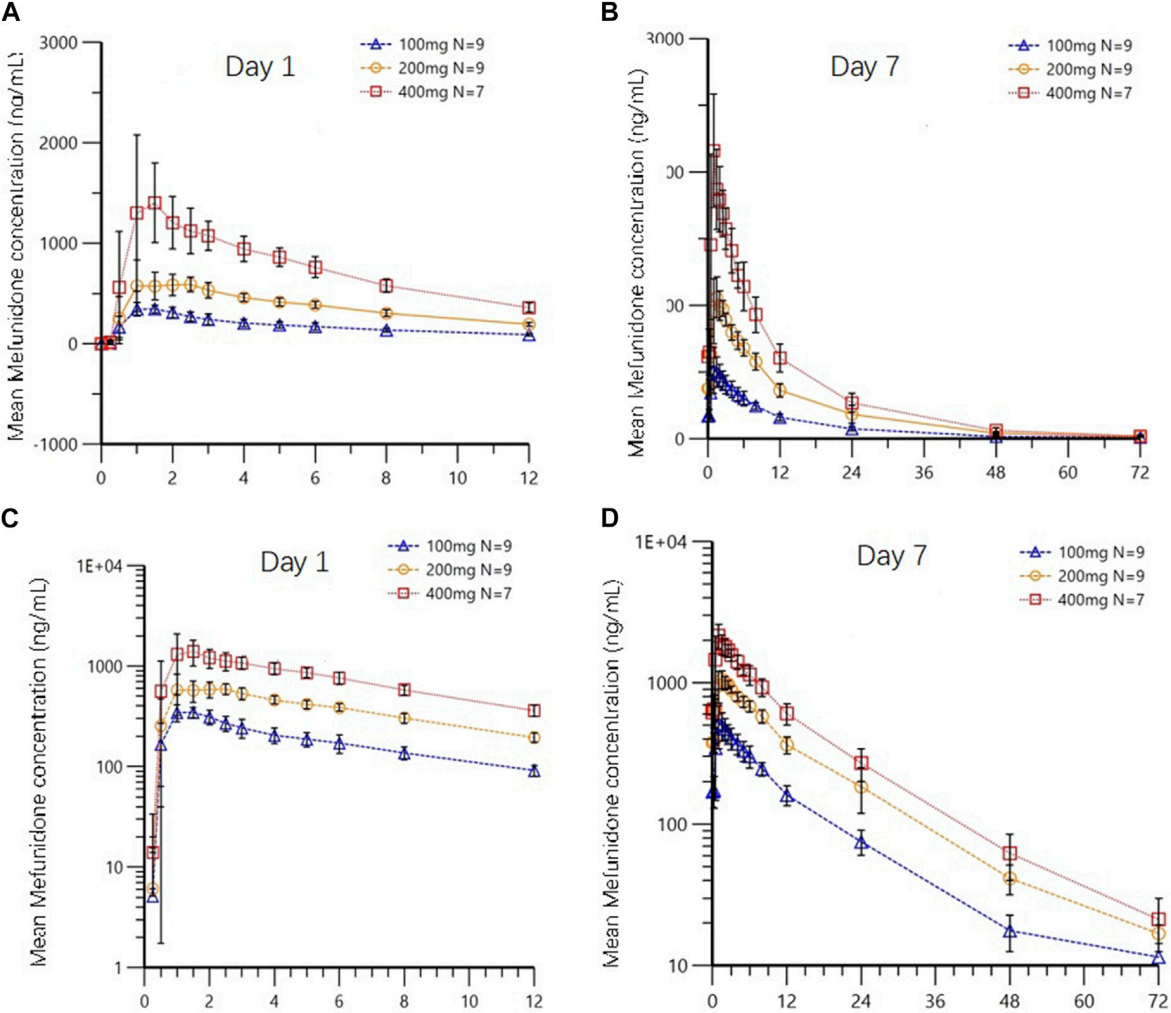
Mean plasma concentrations of mefunidone in the fasted state on day 1 **(A,C)** and day 7 **(B,D)** following multiple-dose administration (Part B). Data are plotted on **(A,B)** linear and **(C,D)** semi-logarithmic scales.

Following multiple-dose administration of mefunidone ranging from 200 to 800 mg daily (as 100 mg, 200 mg, and 400 mg BID), the trough plasma concentrations of mefunidone were similar in each dose cohort before morning administration on D5, D6, and D7, and before evening administration on D5 and D6 (*p* > 0.05) in each dose cohort, respectively. These PK data indicate that a steady state for mefunidone was achieved on day 5 for all doses. Mefunidone was rapidly absorbed, with median T_max_ values ranging from 1.0 to 1.5 h across dose groups, with no dose-dependent trend observed. The mean apparent terminal elimination t_1/2_ was around 12 h (ranging from 11.4 h to 12.3 h), with no dose-dependent trend observed, either. Across dose levels, the accumulation ratio by day 7 was 1.50–1.64 for C_max_ (Rac_C_max_) and 1.66–1.83 for AUC (Rac_AUC_0-tau_), respectively. The dose-normalized exposure parameters (C_max,ss_/D, AUC_0-t,ss_/D, and AUC_0-∞,ss_/D) were similar across dose groups (Table 6). The point estimates and the 90% confidence limits of C_max,ss_, AUC_0-t,ss_, and AUC_0-∞,ss_ were entirely contained within 0.839–1.161, so a dose-proportional increase in exposure between the 100 mg BID and 400 mg BID dosing regimens was concluded (Table 3; Table 6; Figure 4).

**TABLE 6:** Pharmacokinetic parameters of mefunidone by treatment group in Part B.

Parameter	Mefunidone					
	(B1) 100 mg		(B2) 200 mg		(B3) 400 mg	
	Day 1 (n = 9)	Day 7 (n = 9)	Day 1 (n = 9)	Day 7 (n = 9)	Day 1 (n = 9)	Day 7 (n = 7)
AUC_0-τ_ (h*ng/mL)	2,093.9 (1.1)^a^	3,615.37 (1.2)	4,369.7 (1.1)^a^	7,995.25 (1.1)	8,795.8 (1.1)^a^	13,892.97 (1.2)
AUC_0-t,ss/Dc_ (h*ng/mL/mg)	-	59.3 (16.7)	-	69.3 (16.5)	-	57.4 (18.8)
AUC_0-∞,SS_ (h*ng/mL)	-	6,202.8 (15.9)	-	14,193.4 (15.8)	-	23,331.5 (19.3)
Rac_AUC 0-τ	-	1.73 (1.13)	-	1.83 (1.08)	-	1.65 (1.11)
AUC_0-∞,ss/Dc_ (h*ng/mL)	-	62.0 (15.9)	-	71.0 (15.8)	-	58.3 (19.3)
C_max_ (ng/mL)	375.2 (1.12)	554.0 (1.3)	690.2 (1.2)	1,118.4 (1.1)	1,488.2 (1.4)	2,158.6 (1.2)
C_max,ss/Dc_ (ng/mL/mg)	-	5.5 (24.6)	-	5.6 (12.9)	-	5.4 (19.9)
Rac_C max		1.48 (1.23)		1.62 (1.18)		1.60 (1.28)
t_max_ ^b^ (hr)	1.0 (0.5, 1.5)	1.0 (0.5, 2.0)	1.5 (1.0, 2.5)	1.5 (1.0, 2.0)	1.5 (0.5, 3.0)	1.0 (0.5, 2.0)
t_1/2_ (hr)	-	11.41 (1.13)	5.91 (1.13)	12.29 (1.13)	5.65 (1.13)	12.02 (1.13)
CL/F (mL/min)	-	27.7 (1.2)	33.10 (1.09)	25.0 (1.1)	34.00 (1.13)	28.8 (1.2)
V_z_/F (L)	-	455.2 (1.2)	282.3 (1.1)	443.4 (1.1)	277.3 (1.2)	499.1 (1.1)

AUC_0-τ_, area under the concentration–time curve (AUC) during the dosing interval τ); AUC_0-∞_, AUC extrapolated to infinity; C_max_, maximum observed plasma concentration; CL/F, apparent total clearance; CV%, coefficient of variation; RA_AUC_, accumulation ratio based on AUC_0-τ_; t_max_, time to maximum concentration; t_1/2_, apparent terminal elimination half-life; V_z_/F, apparent volume of distribution during the terminal elimination phase.

Data are geometric means (geometric CV%), unless otherwise indicated.

^a^ τ = 12 h.

^b^ Median (range).

^c^ Dose-normalized exposure parameters.

## 4 Discussion

This first-in-human Phase 1 study assessed the safety and pharmacokinetic characteristics of mefunidone as ascending single oral doses of up to 800 mg and multiple oral doses of up to 400 mg twice daily in healthy adult volunteers. The single doses were well-tolerated and exhibited a favorable safety profile without SAEs or dose-limiting adverse events. All TEAEs were mild and reversible. The most common adverse events were investigations (abnormal laboratory results) (24% in mefunidone-treated subjects, accounting for 41.7% of all AEs in Part A), followed by gastrointestinal disorders and nervous system disorders (22% and 18% in mefunidone-treated subjects, respectively). In preclinical studies, the possible target organs of toxicity for mefunidone are the heart, liver, adrenal gland, gastrointestinal tract, and central nervous system (unpublished data). In this study, TEAEs indicating damage to these systems or organs were adverse events of special interest (AESI). We observed no signs suggesting that mefunidone-treated subjects were at higher risk for cardiac, hepatic, or adrenal toxicity compared to placebo-treated subjects. Photosensitivity is commonly reported in pirfenidone-treated patients and is a major reason for discontinuation or dose reduction of this drug (Retnakaran et al., 2006; Boor et al., 2010). The mechanism is thought to result from the structure of pyridone in pirfenidone, a structure that enables resonance stabilization and high skin absorption of UV radiation (Retnakaran et al., 2006; Boor et al., 2010). In this study, the only case of photosensitivity was seen in a placebo-treated subject. None of the mefunidone-treated subjects experienced photosensitivity. This is in accordance with preclinical studies in which mefunidone tested negative for photosensitivity in guinea pigs and other animals. The advantage of reduced photosensitivity with mefunidone may result from the structural modification of this novel compound.

Overall, there was no apparent relationship between mefunidone dose levels and the frequency or severity of TEAEs. In Part A and Part B, some types of adverse events only occurred once, such as hypertriglyceridemia, hyperuricemia, white blood cell decrease, and alanine aminotransferase increase. (Supplementary Tables S2–S4). These uncommon adverse events might be affected by other factors, such as the stimulation of intravenous blood sampling, the hospital setting, or normal fluctuations of hematological and biochemical parameters. However, considering that this is the first-in-human clinical trial of mefunidone, for fear of missing potential safety signals, the investigators preferred to attribute the above adverse events to mefunidone rather than other reasons. Even so, judging from the incidence and severity, the results proved favorable safety profiles for mefunidone.

However, the top two high-dose cohorts (600 mg and 800 mg, A6 and A7, respectively) had relatively higher TEAE incidence rates compared to the lower-dose groups. In these two groups, there were five (83%) and three (75%) mefunidone-treated subjects who experienced TEAEs, respectively. Each of them had at least one nervous system disorder (mostly dizziness) and at least one gastrointestinal disorder (mostly nausea). The comorbidity of nervous system disorders and gastrointestinal disorders was relatively specific for TEAEs in high-dosage groups. In contrast, there were a total of 40 mefunidone-treated subjects in the pooled four lower-dose cohorts (25 mg–200 mg, A1 to A4), only 15 (15/40, 38%) of whom experienced TEAEs, and no case of the comorbidity of nervous system–gastrointestinal disorders was observed.

The safety signals involving the gastrointestinal and neurological systems are worth noting. In preclinical trials, mefunidone cannot easily penetrate the blood–brain barrier, resulting in very low drug concentrations in the brain. Therefore, whether the neurological adverse events observed in this first-in-human trial are due to the direct effect of mefunidone on the central nervous system or to reactions induced by gastrointestinal discomfort requires further clinical observation and investigation.

On the other hand, in the MAD study, adverse events of the gastrointestinal and nervous systems were less and milder than in the A6 and A7 cohorts in the SAD study. None of the mefunidone-treated subjects in the MAD study developed TEAEs of gastrointestinal disorders, while two subjects (in cohorts B2 and B3 each) experienced dizziness. It is notable that the onset of symptoms of nervous system disorders and gastrointestinal disorders was generally consistent with T_max_ in cohorts A6 and A7 (about 0.8–2.5 h post-dose). Moreover, C_max_ for the mefunidone 600 mg single dose was already higher than C _max,ss_ for 400 mg twice daily on day 7 (2644.8 vs. 2158.6 ng/mL). Because of the more-than-proportional increases in C_max_ from the 600 mg to the 800 mg single dose, C_max_ for the mefunidone 800 mg single dose was as high as 4,075.5 ng/mL, approximately twice the C_max,ss_ for 800 mg daily administered at 400 mg twice daily on day 7 (4,075.5 vs. 2,158.6 ng/mL). It is, therefore, reasonable to assume that high peak plasma concentration (C_max_) of mefunidone contributes significantly to adverse events in the gastrointestinal and nervous systems. We recommend that high-dose mefunidone be administered twice daily. Blunting the surge in plasma concentration may reduce nervous and (or) gastrointestinal system disorders and enhance patient adherence.

In Cohort B3 (400 mg BID) of the MAD study, two out of nine mefunidone-treated subjects withdrew due to TEAEs, one with a maculo-papular rash and the other with tonsillitis. Their symptoms began on D4 and D3, and the investigators terminated their participation on D6 and D4, respectively. Although their data were excluded from the final PK analysis, their AUC_0-t_ on Day 1 were the two highest in this group (9,662.4 and 10,945.8 h*ng/mL, respectively, compared to an average AUC_0-t_ of 8,795.8 h*ng/mL in the rest of this group). Considering that the moderate accumulative effect was observed at BID doses in the 400 mg cohort (Rac_C_max_ and Rac_AUC_0-tau_ were 1.60 and 1.65 on day 7, respectively, see Table 6), the systemic exposure in these two subjects would still exceed the systemic exposure in the remaining subjects by the time of their withdrawal. These findings, together with the results of preclinical chronic toxicology studies (see the section on dose selection), suggest that the regimen of mefunidone 400 mg BID should be used with caution because such a dose may result in systemic exposure that triggers intolerance.

In the SAD study, over the dose range of 25 mg–800 mg, systemic exposure to mefunidone (based on AUC) was not completely linearly related to increases in administered doses. The lack of proportionality from 25 mg to 50 mg may be affected by the small sample size of Cohort A1 (four mefunidone-treated subjects compared to eight mefunidone-treated subjects in cohorts A2, A4, and A5). Within the single-dose range of 50 mg–600 mg and the multiple-dose range of 100 mg BID to 400 mg BID by day 7, mefunidone showed good proportionality. High-dose mefunidone resulted in excessive systemic exposure that affected proportionality. From 600 mg to 800 mg single doses, more-than-proportional increases in C_max_ and AUC were observed. Apparent total plasma clearance, CL/F, was the lowest in the 800-mg dose group (386.1 mL/min) compared to 465.2–540.9 mL/min in the other SAD groups. These data indicate that mefunidone has saturable metabolic clearance processes, and high doses will limit the capacity of metabolic and/or excretory clearance pathways. The dosage ranging from 600 mg to 800 mg, however, may have minor significance in clinical use. In preclinical studies, the anti‐fibrotic effect of mefunidone was estimated to be 20‐fold stronger than that of pirfenidone (Wu, 2013; Liu et al., 2015; Jiang et al., 2021), so the proportional dose range of mefunidone may well cover its therapeutic margin.

High-fat-fed conditions led to a delay in T_max_ by approximately 1 hour and a reduction in C_max_ by approximately 20% compared to fasted conditions, but it did not significantly affect the systemic exposure of mefunidone. These data suggest that mefunidone may be given without regard to food. Given the tolerability profile observed in this study, such as possible relevance to higher C_max_ and higher incidence of nervous system and gastrointestinal adverse events, administration with food is recommended more for high doses of mefunidone or in patients with underlying conditions because the effect of food may help blunt the C_max_ effect and, therefore, improve tolerability.

## 5 Conclusion

The present study outlined the pharmacokinetics and safety profiles of mefunidone in healthy Chinese subjects. The ascending single oral doses of up to 800 mg and multiple oral doses up f to 400 mg twice daily were well-tolerated in healthy adult volunteers. The predicted adverse events of special interest from preclinical studies did not cause serious or dose-limiting adverse events. There were no signs suggesting that mefunidone-treated subjects were at higher risk of cardiac, hepatic, adrenal toxicity, or photosensitivity compared to placebo-treated subjects. The comorbidity of nervous system disorders and gastrointestinal disorders was relatively specific to high-dosage mefunidone and was thought to be related to an increase in plasma concentration. Mefunidone behaved with ideal dose proportionality within a single dose range of 50 mg–600 mg and a multiple dose range of 100 mg BID to 400 mg BID by day 7, while high-dose mefunidone resulted in excessive systemic exposure that affected proportionality, suggesting saturable metabolic clearance processes of mefunidone. High-fat-fed conditions led to a delay in T_max_ and a reduction in C_max_ compared to those in fasted conditions, but it did not significantly affect the systemic exposure of mefunidone. Administration with food is recommended for high-dose mefunidone or for patients with underlying conditions.

Mefunidone exhibited favorable pharmacokinetics and safety profiles. The present study has informed and supported the further development of clinical studies with mefunidone.

## Data Availability

The raw data supporting the conclusion of this article will be made available by the authors, without undue reservation.
