# Supplementary material for: A phase I, randomized study to evaluate the safety, tolerability, and pharmacokinetics of mefunidone in healthy subjects
Source: Front Pharmacol. 2024 Jun 12;15:1414066. doi: 10.3389/fphar.2024.1414066 (PMC11199538; doi:10.3389/fphar.2024.1414066)
Supplement: Supplementary file 1 [file Table1.pdf]

Supplementary Table 1. Treatment-emergent adverse events in Part A.

Part A: Groups A1–A6

		Mefunidone							
System organ class<br>Preferred term	Pooled Placebo [a]<br>(n = 14)	A1 (25 mg)<br>(n = 4)	A2 (50 mg)<br>(n = 8)	A3 (100 mg)<br>Fasted<br>(n = 12)	A4 (200 mg)<br>(n = 8)	A5 (400 mg)<br>(n = 8)	A6 (600 mg)<br>(n = 6)	A7 (800mg)<br>(n = 4)	Active total<br>(n = 50)
**Overall total**	**4[5]**	**1[1]**	**2[3]**	**6[12]**	**3[5]**	**3[4]**	**5[15]**	**3[8]**	**23[48]**
**Metabolism and nutrition disorders**	**0**	**0**	**1[1]**	**0**	**0**	**1[1]**	**1[1]**	**0**	**3[3]**
Hypertriglyceridemia	0	0	1[1]	0	0	0	0	0	1[1]
Hyperuricemia	0	0	0	0	0	1[1]	0	0	1[1]
Anorexia	0	0	0	0	0	0	1[1]	0	1[1]
**Ear and labyrinth disorders**	**1[1]**	**0**	**0**	**0**	**0**	**0**	**0**	**0**	**0**
Ear pain	1[1]	0	0	0	0	0	0	0	0
**Investigations**	**4[4]**	**1[1]**	**2[2]**	**3[8]**	**3[5]**	**1[2]**	**1[1]**	**1[1]**	**12[20]**
White blood cell decreased	0	0	0	1[1]	0	0	0	0	1[1]
Alanine aminotransferase increased	0	0	0	0	0	1[1]	0	0	1[1]
Serum amylase increased	0	1[1]	0	0	1[1]	0	0	0	2[2]
Hematocrit decreased	0	0	0	2[2]	0	0	0	0	2[2]

TEAE, treatment-emergent adverse event. Data are no. of subjects [no. of TEAEs].

Placebo is pooled across all Part A treatment periods.

Supplementary Table 1. Treatment-emergent adverse events in Part A. (Continued)

Part A: Groups A1–A6									
		Mefunidone							
System organ class<br>Preferred term	Pooled Placebo [a]<br>(n = 14)	A1 (25 mg)<br>(n = 4)	A2 (50 mg)<br>(n = 8)	A3 (100 mg) Fasted<br>(n = 12)	A4 (200 mg)<br>(n = 8)	A5 (400 mg)<br>(n = 8)	A6 (600 mg)<br>(n = 6)	A7 (800mg)<br>(n = 4)	Active total<br>(n = 50)
Lymphocyte count decreased	1[1]	0	0	0	0	0	0	0	0
Urine leukocyte increased	0	0	1[1]	0	0	0	0	0	1[1]
Blood in the urine	0	0	0	0	2[2]	0	1[1]	0	3[3]
Mean corpuscular volume decreased	0	0	0	1[1]	0	0	0	0	1[1]
Mean corpuscular hemoglobin decreased	0	0	0	1[1]	0	0	0	0	1[1]
Stool occult blood positive	1[1]	0	0	0	1[1]	0	0	0	1[1]
Basophil count increased	0	0	0	0	1[1]	0	0	1[1]	2[2]
Blood corticotrophin decreased	1[1]	0	0	0	0	0	0	0	0
Blood corticotrophin increased	1[1]	0	1[1]	0	0	0	0	0	1[1]

TEAE, treatment-emergent adverse event. Data are no. of subjects [no. of TEAEs].

Placebo is pooled across all Part A treatment periods.

Supplementary Table 1. Treatment-emergent adverse events in Part A. (Continued)

Part A: Groups A1–A6

	Mefunidone								
System organ class<br>Preferred term	Pooled Placebo [a]<br>(n = 14)	A1 (25 mg)<br>(n = 4)	A2 (50 mg)<br>(n = 8)	A3 (100 mg)<br>Fasted<br>(n = 12)	A4 (200 mg)<br>(n = 8)	A5 (400 mg)<br>(n = 8)	A6 (600 mg)<br>(n = 6)	A7 (800mg)<br>(n = 4)	Active total<br>(n = 50)
Hemoglobin decreased	0	0	0	2[2]	0	0	0	0	2[2]
CK-MB （myoglobin） increased	0	0	0	1[1]	0	0	0	0	1[1]
CPK (creatine phosphokinase) increased	0	0	0	0	0	1[1]	0	0	1[1]
**Nervous system disorders**	**0**	**0**	**0**	**1[1]**	**0**	**0**	**5[5]**	**3[3]**	**9[9]**
Headache	0	0	0	1[1]	0	0	0	0	1[1]
Dizziness	0	0	0	0	0	0	5[5]	3[3]	8[8]
**General disorders and administration site conditions**	**0**	**0**	**0**	**0**	**0**	**1[1]**	**0**	**0**	**1[1]**
Non-cardiac chest pain	0	0	0	0	0	1[1]	0	0	1[1]
**Gastrointestinal disorders**	**0**	**0**	**0**	**3[3]**	**0**	**0**	**5[7]**	**3[4]**	**11[14]**
Nausea	0	0	0	0	0	0	2[2]	3[3]	5[5]

TEAE, treatment-emergent adverse event. Data are no. of subjects [no. of TEAEs].

Placebo is pooled across all Part A treatment periods.

Supplementary Table 1. Treatment-emergent adverse events in Part A. (Continued)

Part A: Groups A1–A6									
	Mefunidone								
System organ class<br>Preferred term	Pooled Placebo [a]<br>(n = 14)	A1 (25 mg)<br>(n = 4)	A2 (50 mg)<br>(n = 8)	A3 (100 mg)<br>Fasted<br>(n = 12)	A4 (200 mg)<br>(n = 8)	A5 (400 mg)<br>(n = 8)	A6 (600 mg)<br>(n = 6)	A7 (800mg)<br>(n = 4)	Active total<br>(n = 50)
Diarrhea	0	0	0	0	0	0	1[1]	0	1[1]
Abdominal distension	0	0	0	0	0	0	3[3]	0	3[3]
Vomiting	0	0	0	0	0	0	1[1]	1[1]	2[2]
Abdominal pain	0	0	0	3[3]	0	0	0	0	3[3]
**Vascular disorders**	**0**	**0**	**0**	**0**	**0**	**0**	**1[1]**	**0**	**1[1]**
Hypotension	0	0	0	0	0	0	1[1]	0	1[1]

TEAE, treatment-emergent adverse event. Data are no. of subjects [no. of TEAEs].

Placebo is pooled across all Part A treatment periods.

Supplementary Table 2. Treatment-emergent adverse events in food effect study (A3 in Part A).

Part A: A3 (food effect)				
System organ class Preferred term	Fasted (Period 1)		High-fat fed (Period 2)	
	Active (n = 12)	Placebo (n=2)	Active (n = 11)	Placebo (n=2)
**Overall total**	**6[12]**	**0**	**6[8]**	**0**
**Metabolism and nutrition disorders**	**0**	**0**	**1[1]**	**0**
Hypertriglyceridemia	0	0	1[1]	0
**Investigations**	**3[8]**	**0**	**4[5]**	**0**
White blood cell decreased	1[1]	1[1]	0	0
Hematocrit decreased	2[2]	0	1[1]	0

TEAE, treatment-emergent adverse event. Data are no. of subjects [no. of TEAEs].

Supplementary Table 2. Treatment-emergent adverse events in food effect study (A3 in Part A). (Continued)

Part A: A3 (food effect)				
System organ class Preferred term	Fasted (Period 1)		High-fat fed (Period 2)	
	Active (n = 12)	Placebo (n=2)	Active (n = 11)	Placebo (n=2)
Mean corpuscular volume decreased	1[1]	0	0	0
Mean corpuscular hemoglobin decreased	1[1]	0	0	0
Hemoglobin decreased	2[2]	0	1[1]	0
CK-MB（myoglobin） increased	1[1]	0	0	0
Neutrophil count decreased	0	0	1[1]	0
Total bile acid increased	0	0	1[1]	0
**Nervous system disorders**	**1[1]**	**0**	**0**	**0**
Headache	1[1]	0	0	0
**General disorders and administration site conditions**	**0**	**0**	**1[1]**	**0**
Fatigue	0	0	1[1]	0
**Gastrointestinal disorders**	**3[3]**	**0**	**1[1]**	**0**
Diarrhea	0	0	1[1]	0
Abdominal pain	3[3]	0	0	0

TEAE, treatment-emergent adverse event. Data are no. of subjects [no. of TEAEs].

Supplementary Table 3. Treatment-emergent adverse events in Part B.

Part B: Groups B1-B3					
			Mefunidone		
System organ class Preferred term	Pooled placebo (n = 9)	B1 (100 mg) (n = 9)	B2 (200 mg) (n = 9)	B3 (400 mg) (n = 9)	Active total (n = 27)
**Overall total**	**8[17]**	**7[11]**	**9[18]**	**6[15]**	**22[44]**
**Investigations**	**7[12]**	**5[6]**	**6[10]**	**3[6]**	**14[22]**
Total bile acid increased	1[1]	2[2]	2[2]	0	4[4]
Alanine aminotransferase increased	1[1]	1[1]	1[1]	1[1]	3[3]
Cortisol hormone increased	0	0	1[1]	2[2]	3[3]
Blood corticotrophin increased	2[2]	0	1[1]	1[1]	2[2]
Hemoglobin decreased	0	0	2[2]	0	2[2]
Neutrophil count decreased	2[2]	0	2[2]	0	2[2]
Urine leukocyte increased	1[1]	1[1]	0	0	1[1]
Blood in urine	0	1[1]	0	0	1[1]
Urinary retinal binding protein increased	0	0	0	1[1]	1[1]
Stool occult blood positive	2[2]	0	1[1]	0	1[1]
CK-MB（myoglobin） increased	0	1[1]	0	0	1[1]
Fibrinogen decreased	0	0	0	1[1]	1[1]
Serum amylase increased	1[1]	0	0	0	0
Aspartate aminotransferase increased	1[1]	0	0	0	0
Blood corticotrophin decreased	1[1]	0	0	0	0
**Metabolism and nutrition disorders**	**0**	**3[4]**	**4[5]**	**3[5]**	**10[14]**
Hypertriglyceridemia	0	2[2]	4[4]	0	6[6]

TEAE, treatment-emergent adverse event. Data are no. of subjects [no. of TEAEs].

Placebo is pooled across all Part B treatment periods.

Supplementary Table 3. Treatment-emergent adverse events in Part B. (Continued)

Part B: Groups B1-B3					
		Mefunidone			
System organ class Preferred term	Pooled placebo (n = 9)	B1 (100 mg) (n = 9)	B2 (200 mg) (n = 9)	B3 (400 mg) (n = 9)	Active total (n = 27)
Hyponatremia	0	2[2]	0	3[3]	5[5]
Hypophosphatemia	0	0	0	1[1]	1[1]
Hyperuricemia	0	0	1[1]	0	1[1]
Anorexia	0	0	0	1[1]	1[1]
**Nervous system disorders**	**0**	**0**	**1[1]**	**1[1]**	**2[2]**
Dizziness	0	0	1[1]	1[1]	2[2]
**Skin and subcutaneous tissue disorders**	**1[1]**	**0**	**0**	**2[2]**	**2[2]**
Rash maculo-papular	0	0	0	2[2]	2[2]
Photosensitivity	1[1]	0	0	0	0
**Cardiac disorders**	**0**	**1[1]**	**1[1]**	**0**	**2[2]**
Conduction disorder	0	0	1[1]	0	1[1]
Sinus tachycardia	0	1[1]	0	0	1[1]
**Hepatobiliary disorders**	**0**	**0**	**1[1]**	**0**	**1[1]**
Blood bilirubin increased	0	0	1[1]	0	1[1]
**Infections and infestations**	**1[2]**	**0**	**0**	**1[1]**	**1[1]**
Tonsillitis	0	0	0	1[1]	1[1]
Gum infection	1[2]	0	0	0	0

TEAE, treatment-emergent adverse event. Data are no. of subjects [no. of TEAEs].

Placebo is pooled across all Part B treatment periods.

Supplementary Table 3. Treatment-emergent adverse events in Part B. (Continued)

Part B: Groups B1-B3					
		Mefunidone			
System organ class Preferred term	Pooled placebo (n = 9)	B1 (100 mg) (n = 9)	B2 (200 mg) (n = 9)	B3 (400 mg) (n = 9)	Active total (n = 27)
**Respiratory, thoracic and mediastinal disorders**	**1[1]**	**0**	**0**	**0**	**0**
Epistaxis	1[1]	0	0	0	0
**Gastrointestinal disorders**	**1[1]**	**0**	**0**	**0**	**0**
Mucositis oral	1[1]	0	0	0	0

TEAE, treatment-emergent adverse event. Data are no. of subjects [no. of TEAEs].

Placebo is pooled across all Part B treatment periods.

Supplementary Table 4. Treatment-emergent adverse events occurring in ≥2 subjects in total.

Part A: Period 1, Groups A1–A6									
				Mefunidone					
System organ class Preferred term	Pooled Placebo [a] (n = 14)	A1 (25 mg) (n = 4)	A2 (50 mg) (n = 8)	A3 (100 mg) Fasted (n = 12)	A4 (200 mg) (n = 8)	A5 (400 mg) (n = 8)	A6 (600 mg) (n = 6)	A7 (800mg) (n = 4)	Active total (n = 50)
Serum amylase increased	0	1[1]	0	0	1[1]	0	0	0	2[2]
Hematocrit decreased	0	0	0	2[2]	0	0	0	0	2[2]
Blood in the urine	0	0	0	0	1[1]	0	1[1]	0	2[2]

TEAE, treatment-emergent adverse event. Data are no. of subjects (percent of subjects) [no. of TEAEs].

Placebo is pooled across all Part A and Part B treatment periods.

Supplementary Table 4. Treatment-emergent adverse events occurring in ≥2 subjects in total. (Continued)

Part A: Period 1, Groups A1–A6

		Mefunidone							
System organ class Preferred term	Pooled Placebo [a] (n = 14)	A1 (25 mg) (n = 4)	A2 (50 mg) (n = 8)	A3 (100 mg) Fasted (n = 12)	A4 (200 mg) (n = 8)	A5 (400 mg) (n = 8)	A6 (600 mg) (n = 6)	A7 (800mg) (n = 4)	Active total (n = 50)
Stool occult blood positive	1[1]	0	0	0	1[1]	0	0	0	1[1]
Basophil count increased	0	0	0	0	1[1]	0	0	1[1]	2[2]
Blood corticotrophin increased	1[1]	0	1[1]	0	0	0	0	0	1[1]
Hemoglobin decreased	0	0	0	2[2]	0	0	0	0	2[2]
Dizziness	0	0	0	0	0	0	5[5]	3[3]	8[8]
Nausea	0	0	0	0	0	0	2[2]	3[3]	5[5]
Abdominal distension	0	0	0	0	0	0	3[3]	0	3[3]
Vomiting	0	0	0	0	0	0	1[1]	1[1]	2[2]
Abdominal pain	0	0	0	3[3]	0	0	0	0	3[3]

Part A: Period 1-2, Group A3 (food effect)

System organ class Preferred term	Fasted (Period 1)		High-fat fed (Period 2)	
	Active (n = 12)	Placebo (n=2)	Active (n = 11)	Placebo (n=2)
Hematocrit decreased	2[2]	0	1[1]	0
Hemoglobin decreased	2[2]	0	1[1]	0
Abdominal pain	3[3]	0	0	0

TEAE, treatment-emergent adverse event. Data are no. of subjects (percent of subjects) [no. of TEAEs].

Placebo is pooled across all Part A and Part B treatment periods.

Supplementary Table 4. Treatment-emergent adverse events occurring in ≥2 subjects in total. (Continued)

Part B: Groups B1-B3					
		Mefunidone			
System organ class<br>Preferred term	Pooled placebo<br>(n = 9)	B1 (100 mg)<br>(n = 9)	B2 (200 mg)<br>(n = 9)	B3 (400 mg)<br>(n = 9)	Active total<br>(n = 27)
Total bile acid increased	1[1]	2[2]	2[2]	0	4[4]
Alanine aminotransferase increased	1[1]	1[1]	1[1]	1[1]	3[3]
cortisol hormone increased	0	0	1[1]	2[2]	3[3]
Blood corticotrophin increased	2[2]	0	1[1]	1[1]	2[2]
Hemoglobin decreased	0	0	2[2]	0	2[2]
Neutrophil count decreased	2[2]	0	2[2]	0	2[2]
Urine leukocyte increased	1[1]	1[1]	0	0	1[1]
Stool occult blood positive	2[2]	0	1[1]	0	1[1]
Hypertriglyceridemia	0	2[2]	4[4]	0	6[6]
Hyponatremia	0	2[2]	0	3[3]	5[5]
Dizziness	0	0	1[1]	1[1]	2[2]
Rash maculo-papular	0	0	0	2[2]	2[2]

TEAE, treatment-emergent adverse event. Data are no. of subjects (percent of subjects) [no. of TEAEs].

Placebo is pooled across all Part A and Part B treatment periods.

Supplementary Table 5. Plasma mefunidone concentrations-time profiles of individual subjects in Part A (Period 1; single doses administered in the fasting state).

Subject ID	Dosage	0h ng/mL	0.25h ng/mL	0.5h ng/mL	1h ng/mL	1.5h ng/mL	2h ng/mL	2.5h ng/mL	3h ng/mL	4h ng/mL	5h ng/mL	6h ng/mL	8h ng/mL	12h ng/mL	24h ng/mL	48h ng/mL	72h ng/mL
101	25mg	BLQ	BLQ	BLQ	34.9	44.9	65.7	65.5	51.2	44.0	45.6	41.5	35.2	20.8	11.0	BLQ	BLQ
102	Placebo	BLQ	BLQ	BLQ	BLQ	BLQ	BLQ	BLQ	BLQ	BLQ	BLQ	BLQ	BLQ	BLQ	BLQ	BLQ	BLQ
103	25mg	BLQ	BLQ	14.0	58.2	66.8	63.1	59.3	55.2	48.8	46.1	43.3	32.6	19.7	BLQ	BLQ	BLQ
104	25mg	BLQ	BLQ	BLQ	14.3	43.9	56.0	48.8	47.9	40.9	36.6	36.4	28.2	19.6	BLQ	BLQ	BLQ
105	25mg	BLQ	BLQ	BLQ	12.7	38.6	67.6	70.6	66.3	55.8	47.2	42.3	33.5	22.5	10.9	BLQ	BLQ
106	Placebo	BLQ	BLQ	BLQ	BLQ	BLQ	BLQ	BLQ	BLQ	BLQ	BLQ	BLQ	BLQ	BLQ	BLQ	BLQ	BLQ
201	50mg	BLQ	BLQ	126.0	240.0	201.0	172.0	164.0	147.0	139.0	117.0	109.0	81.5	44.8	13.3	BLQ	BLQ
202	50mg	BLQ	BLQ	33.4	162.0	160.0	136.0	123.0	109.0	96.1	87.8	81.1	60.0	37.0	16.5	BLQ	BLQ
203	Placebo	BLQ	BLQ	BLQ	BLQ	BLQ	BLQ	BLQ	BLQ	BLQ	BLQ	BLQ	BLQ	BLQ	BLQ	BLQ	BLQ
204	50mg	BLQ	BLQ	BLQ	19.3	47.7	66.8	89.9	108.0	97.1	82.6	79.3	64.1	41.4	22.0	BLQ	BLQ
205	50mg	BLQ	BLQ	BLQ	46.8	183.0	203.0	168.0	150.0	131.0	116.0	107.0	85.0	50.1	21.6	BLQ	BLQ
206	50mg	BLQ	BLQ	21.8	135.0	169.0	145.0	125.0	121.0	96.3	84.0	81.5	66.2	40.1	16.1	BLQ	BLQ
207	50mg	BLQ	BLQ	15.6	216.0	191.0	151.0	132.0	107.0	106.0	90.3	84.9	72.5	47.4	20.2	BLQ	BLQ
208	50mg	BLQ	BLQ	18.8	147.0	142.0	138.0	134.0	133.0	122.0	108.0	97.0	73.0	45.5	16.6	BLQ	BLQ
209	50mg	BLQ	BLQ	24.6	135.0	130.0	113.0	99.2	87.7	75.7	68.5	62.1	56.3	34.0	14.0	BLQ	BLQ
210	Placebo	BLQ	BLQ	BLQ	BLQ	BLQ	BLQ	BLQ	BLQ	BLQ	BLQ	BLQ	BLQ	BLQ	BLQ	BLQ	BLQ
301	100mg	BLQ	14.1	184.0	486.0	387.0	333.0	331.0	259.0	252.0	205.0	165.0	139.0	95.7	41.3	BLQ	BLQ
302	100mg	BLQ	BLQ	41.6	358.0	300.0	312.0	220.0	239.0	191.0	191.0	148.0	134.0	72.4	27.3	BLQ	BLQ
303	100mg	BLQ	23.8	373.0	491.0	379.0	366.0	324.0	288.0	260.0	216.0	196.0	142.0	95.8	39.8	BLQ	BLQ
304	100mg	BLQ	10.3	84.6	260.0	330.0	340.0	337.0	350.0	274.0	273.0	225.0	181.0	124.0	47.0	10.6	BLQ
305	100mg	BLQ	14.9	144.0	414.0	331.0	315.0	287.0	235.0	223.0	182.0	179.0	139.0	81.7	35.4	BLQ	BLQ

BLQ, below the limit of quantification.

Supplementary Table 5. Plasma mefunidone concentrations-time profiles of individual subjects in Part A (Period 1; single doses administered in the fasting state). (Continued)

Subject ID	Dosage	0h ng/mL	0.25h ng/mL	0.5h ng/mL	1h ng/mL	1.5h ng/mL	2h ng/mL	2.5h ng/mL	3h ng/mL	4h ng/mL	5h ng/mL	6h ng/mL	8h ng/mL	12h ng/mL	24h ng/mL	48h ng/mL	72h ng/mL
306	100mg	BLQ	BLQ	70.0	357.0	330.0	350.0	295.0	251.0	219.0	185.0	171.0	143.0	74.5	32.7	BLQ	BLQ
307	100mg	BLQ	BLQ	BLQ	105.0	306.0	313.0	293.0	304.0	266.0	210.0	195.0	136.0	82.5	33.7	BLQ	BLQ
308	100mg	BLQ	BLQ	145.0	435.0	443.0	363.0	367.0	316.0	255.0	217.0	196.0	158.0	98.8	31.3	BLQ	BLQ
309	100mg	BLQ	BLQ	41.7	220.0	292.0	314.0	267.0	248.0	178.0	168.0	150.0	98.8	66.7	22.1	BLQ	BLQ
310	Placebo	BLQ	BLQ	BLQ	BLQ	BLQ	BLQ	BLQ	BLQ	BLQ	BLQ	BLQ	BLQ	BLQ	BLQ	BLQ	BLQ
311	100mg	BLQ	BLQ	15.0	267.0	298.0	289.0	314.0	238.0	231.0	227.0	170.0	148.0	95.0	31.2	BLQ	BLQ
312	Placebo	BLQ	BLQ	BLQ	BLQ	BLQ	BLQ	BLQ	BLQ	BLQ	BLQ	BLQ	BLQ	BLQ	BLQ	BLQ	BLQ
313	100mg	BLQ	BLQ	61.8	465.0	421.0	390.0	280.0	316.0	284.0	272.0	241.0	188.0	111.0	48.0	BLQ	BLQ
314	100mg	BLQ	12.5	295.0	371.0	315.0	283.0	258.0	255.0	191.0	172.0	179.0	107.0	66.2	23.7	BLQ	BLQ
401	200mg	BLQ	25.3	280.0	596.0	436.0	348.0	340.0	393.0	305.0	289.0	258.0	189.0	135.0	57.2	11.0	BLQ
402	Placebo	BLQ	BLQ	BLQ	BLQ	BLQ	BLQ	BLQ	BLQ	BLQ	BLQ	BLQ	BLQ	BLQ	BLQ	BLQ	BLQ
403	200mg	BLQ	BLQ	203.0	643.0	606.0	602.0	513.0	483.0	459.0	387.0	364.0	261.0	187.0	64.1	12.7	BLQ
404	200mg	BLQ	BLQ	167.0	692.0	766.0	707.0	704.0	608.0	498.0	416.0	408.0	323.0	186.0	71.8	14.3	BLQ
405	200mg	BLQ	BLQ	63.3	570.0	570.0	476.0	450.0	396.0	370.0	340.0	259.0	241.0	154.0	65.5	12.9	BLQ
406	200mg	BLQ	BLQ	288.0	762.0	596.0	505.0	455.0	379.0	346.0	309.0	284.0	239.0	140.0	56.5	13.7	BLQ
407	200mg	BLQ	BLQ	229.0	734.0	476.0	490.0	483.0	448.0	378.0	348.0	309.0	235.0	154.0	61.6	10.5	BLQ
408	Placebo	BLQ	BLQ	BLQ	BLQ	BLQ	BLQ	BLQ	BLQ	BLQ	BLQ	BLQ	BLQ	BLQ	BLQ	BLQ	BLQ
409	200mg	BLQ	BLQ	111.0	1090.0	941.0	615.0	698.0	647.0	542.0	464.0	423.0	314.0	229.0	72.6	12.4	BLQ
410	200mg	BLQ	11.7	744.0	819.0	648.0	517.0	488.0	408.0	367.0	310.0	274.0	222.0	160.0	70.3	12.7	BLQ

BLQ, below the limit of quantification.

Supplementary Table 5. Plasma mefunidone concentrations-time profiles of individual subjects in Part A (Period 1; single doses administered in the fasting state). (Continued)

Subject ID	Dosage	0h ng/mL	0.25h ng/mL	0.5h ng/mL	1h ng/mL	1.5h ng/mL	2h ng/mL	2.5h ng/mL	3h ng/mL	4h ng/mL	5h ng/mL	6h ng/mL	8h ng/mL	12h ng/mL	24h ng/mL	48h ng/mL	72h ng/mL
501	Placebo	BLQ	BLQ	BLQ	BLQ	BLQ	BLQ	BLQ	BLQ	BLQ	BLQ	BLQ	BLQ	BLQ	BLQ	BLQ	BLQ
502	Placebo	BLQ	BLQ	BLQ	BLQ	BLQ	BLQ	BLQ	BLQ	BLQ	BLQ	BLQ	BLQ	BLQ	BLQ	BLQ	BLQ
503	400mg	BLQ	BLQ	1110.0	1350.0	1320.0	1110.0	1020.0	1020.0	733.0	784.0	686.0	447.0	313.0	129.0	18.4	BLQ
504	400mg	BLQ	20.4	1260.0	1740.0	1570.0	1290.0	1220.0	1130.0	953.0	808.0	745.0	590.0	310.0	91.5	13.2	BLQ
505	400mg	BLQ	18.8	1060.0	1910.0	1380.0	1300.0	1110.0	1060.0	824.0	774.0	712.0	543.0	350.0	136.0	22.9	BLQ
506	400mg	BLQ	18.5	2310.0	1960.0	1510.0	1270.0	1190.0	1030.0	918.0	804.0	748.0	577.0	279.0	123.0	28.8	BLQ
507	400mg	BLQ	16.5	1750.0	1820.0	1570.0	1410.0	1320.0	1170.0	1110.0	972.0	858.0	699.0	403.0	173.0	33.3	10.7
508	400mg	BLQ	13.4	1800.0	1730.0	1440.0	1180.0	1070.0	967.0	866.0	832.0	736.0	578.0	365.0	165.0	35.7	11.4
509	400mg	BLQ	30.6	2040.0	1690.0	1540.0	1480.0	1390.0	1120.0	1040.0	806.0	700.0	585.0	332.0	109.0	17.8	BLQ
510	400mg	BLQ	15.8	959.0	2120.0	1440.0	1290.0	1170.0	1190.0	1060.0	912.0	860.0	746.0	491.0	199.0	38.5	10.3
601	600mg	BLQ	65.3	2360.0	2340.0	2250.0	2150.0	1930.0	1820.0	1530.0	1310.0	1190.0	853.0	468.0	159.0	27.8	BLQ
602	600mg	BLQ	60.6	1910.0	2000.0	1820.0	1740.0	1720.0	1500.0	1350.0	1120.0	1010.0	817.0	415.0	164.0	33.7	11.9
603	600mg	BLQ	100.0	3660.0	2830.0	2390.0	2050.0	1700.0	1560.0	1410.0	1300.0	1130.0	943.0	555.0	190.0	34.3	11.0
604	Placebo	BLQ	BLQ	BLQ	BLQ	BLQ	BLQ	BLQ	BLQ	BLQ	BLQ	BLQ	BLQ	BLQ	BLQ	BLQ	BLQ
605	Placebo	BLQ	BLQ	BLQ	BLQ	BLQ	BLQ	BLQ	BLQ	BLQ	BLQ	BLQ	BLQ	BLQ	BLQ	BLQ	BLQ
606	600mg	BLQ	14.0	663.0	2910.0	2360.0	1880.0	1510.0	1450.0	1310.0	1050.0	1060.0	862.0	567.0	182.0	32.5	10.6
607	600mg	BLQ	374.0	3660.0	2710.0	2270.0	1780.0	1600.0	1430.0	1340.0	1210.0	1070.0	823.0	488.0	208.0	41.0	13.0
608	600mg	BLQ	BLQ	53.3	601.0	1860.0	1660.0	1420.0	1260.0	1100.0	1110.0	958.0	724.0	430.0	151.0	27.8	BLQ
701	Placebo	BLQ	BLQ	BLQ	BLQ	BLQ	BLQ	BLQ	BLQ	BLQ	BLQ	BLQ	BLQ	BLQ	BLQ	BLQ	BLQ
702	800mg	BLQ	402.0	3910.0	4460.0	3940.0	4680.0	3430.0	3350.0	2960.0	2450.0	2220.0	1550.0	885.0	372.0	59.3	20.3

BLQ, below the limit of quantification.

Supplementary Table 5. Plasma mefunidone concentrations-time profiles of individual subjects in Part A (Period 1; single doses administered in the fasting state). (Continued)

Subject ID	Dosage	0h ng/mL	0.25h ng/mL	0.5h ng/mL	1h ng/mL	1.5h ng/mL	2h ng/mL	2.5h ng/mL	3h ng/mL	4h ng/mL	5h ng/mL	6h ng/mL	8h ng/mL	12h ng/mL	24h ng/mL	48h ng/mL	72h ng/mL
703	800mg	BLQ	25.3	2020.0	2020.0	2890.0	2920.0	2970.0	2420.0	2380.0	2130.0	1890.0	1330.0	936.0	335.0	51.8	12.4
704	Placebo	BLQ	BLQ	BLQ	BLQ	BLQ	BLQ	BLQ	BLQ	BLQ	BLQ	BLQ	BLQ	BLQ	BLQ	BLQ	BLQ
705	800mg	BLQ	137.0	4010.0	3790.0	3960.0	3170.0	2750.0	2570.0	2270.0	2400.0	2190.0	1540.0	1400.0	538.0	120.0	33.8
706	800mg	BLQ	139.0	4870.0	3690.0	3450.0	2840.0	2610.0	2290.0	2220.0	2340.0	1960.0	1350.0	789.0	251.0	39.1	10.0

BLQ, below the limit of quantification.

Supplementary Table 6. Individual pharmacokinetic parameters of mefunidone in Part A (Period 1; single doses administered in the fasting state).

Subject ID	Dosage	C_{max} ng/mL	T_{max} h	$t_{1/2}$ h	AUC_{0-t} h*ng/mL	$AUC_{0-\infty}$ h*ng/mL	CL/F mL/h	V_z/F mL
101	25mg	65.7	2.0	9.26	633.32	780.29	32039.37	428082.24
103	25mg	66.8	1.5	5.31	466.36	617.33	40497.07	310336.84
104	25mg	56.0	2.0	7.19	375.34	578.75	43196.51	448303.60
105	25mg	70.6	2.5	10.21	649.54	810.03	30863.04	454419.68
201	50mg	240.0	1.0	5.86	1617.33	1729.81	28904.88	244451.76
202	50mg	162.0	1.0	7.61	1247.42	1428.68	34997.32	384466.00
204	50mg	108.0	3.0	9.47	1135.50	1435.99	34819.16	475581.65
205	50mg	203.0	2.0	7.49	1586.05	1819.47	27480.56	296968.16
206	50mg	169.0	1.5	7.71	1279.83	1458.89	34272.62	381181.63
207	50mg	216.0	1.0	8.69	1446.72	1699.96	29412.53	368723.31
208	50mg	147.0	1.0	6.97	1413.30	1580.23	31641.02	318173.24

$AUC_{0-\infty}$, AUC, area under the concentration-time curve (AUC)extrapolated to infinity; C_{max}, maximum observed plasma concentration; CL/ F, apparent total clearance; T_{max}, time to maximum concentration; $t_{1/2}$, apparent terminal elimination half-life; V_z/F, apparent volume of distribution during the terminal elimination phase.

Supplementary Table 6. Individual pharmacokinetic parameters of mefunidone in Part A (Period 1; single doses administered in the fasting state). (Continued)

Subject ID	Dosage	C_{max} ng/mL	T_{max} h	$t_{1/2}$ h	AUC_{0-t} h*ng/mL	$AUC_{0-\infty}$ h*ng/mL	CL/F mL/h	V_z/F mL
209	50mg	135.0	1.0	8.16	1057.08	1221.87	40920.90	481668.99
301	100mg	486.0	1.0	9.08	3114.46	3655.58	27355.41	358419.48
302	100mg	320.0	4.0	7.96	2213.84	2648.09	37763.03	433829.32
303	100mg	372.0	1.5	8.25	3241.79	3705.86	26984.31	321090.34
304	100mg	350.0	3.0	9.89	4133.75	4284.98	23337.35	332954.94
305	100mg	395.0	1.5	7.27	2900.96	3277.45	30511.57	319974.24
306	100mg	261.0	4.0	7.06	2548.80	2889.97	34602.39	352405.56
307	100mg	313.0	2.0	8.22	2744.25	3144.05	31806.15	377332.12
308	100mg	443.0	1.5	6.79	3242.41	3549.05	28176.52	276041.80
309	100mg	186.0	2.5	7.74	1709.99	1947.85	51338.71	573301.39
311	100mg	385.0	2.0	8.14	2832.38	3256.43	30708.44	360719.73
313	100mg	465.0	1.0	7.54	3633.10	4155.01	24067.32	261686.38
314	100mg	371.0	1.0	7.52	2484.91	2741.90	36471.07	395466.65
401	200mg	596.0	1.0	9.96	4828.13	4986.22	40110.56	576448.40
403	200mg	643.0	1.0	9.17	6313.44	6481.54	30856.88	408417.45
404	200mg	766.0	1.5	9.79	7098.50	7300.44	27395.62	386870.83
405	200mg	570.0	1.0	10.08	5492.86	5680.41	35208.74	511894.99
406	200mg	762.0	1.0	10.85	5469.20	5683.72	35188.20	551007.97
407	200mg	734.0	1.0	9.30	5625.71	5766.56	34682.75	465240.80
409	200mg	1090.0	1.0	8.54	7676.53	7829.25	25545.23	314613.37

$AUC_{0-\infty}$, AUC, area under the concentration-time curve (AUC)extrapolated to infinity; C_{max}, maximum observed plasma concentration; CL/ F, apparent total clearance; T_{max}, time to maximum concentration; $t_{1/2}$, apparent terminal elimination half-life; V_z/F, apparent volume of distribution during the terminal elimination phase.

Supplementary Table 6. Individual pharmacokinetic parameters of mefunidone in Part A (Period 1; single doses administered in the fasting state). (Continued)

Subject ID	Dosage	C_{max} ng/mL	T_{max} h	$t_{1/2}$ h	AUC_{0-t} h*ng/mL	$AUC_{0-\infty}$ h*ng/mL	CL/F mL/h	V_z/F mL
410	200mg	819.0	1.0	9.82	5996.77	6176.65	32379.99	458639.65
503	400mg	1350.0	1.0	8.74	12043.27	12275.31	32585.74	410925.60
504	400mg	1740.0	1.0	8.00	12548.90	12701.19	31493.12	363343.63
505	400mg	1910.0	1.0	9.18	13232.37	13535.57	29551.77	391269.13
506	400mg	2310.0	0.5	11.05	13492.44	13951.70	28670.33	457194.60
507	400mg	1820.0	1.0	11.37	16580.48	16755.97	23872.08	391531.16
508	400mg	1800.0	0.5	11.93	14894.25	15090.47	26506.79	456240.31
509	400mg	2040.0	0.5	8.62	13524.36	13745.61	29100.19	361709.83
510	400mg	2120.0	1.0	10.18	17431.28	17582.59	22749.78	334188.80
601	600mg	2360.0	0.5	8.93	19856.84	20215.07	29680.83	382461.56
602	600mg	2000.0	1.0	11.70	18385.96	18586.75	32281.06	544692.63
603	600mg	3660.0	0.5	9.26	22061.86	22208.80	27016.32	360894.65
606	600mg	2910.0	1.0	9.38	19890.40	20033.83	29949.34	405245.12
607	600mg	3660.0	0.5	11.40	21218.05	21431.87	27995.69	460466.38
608	600mg	1860.0	1.5	9.21	15298.92	15668.20	38294.12	508681.16
702	800mg	4680.0	2.0	10.83	39348.02	39665.12	20168.85	315059.21
703	800mg	2970.0	2.5	9.41	32420.81	32589.08	24548.10	333122.65
705	800mg	4010.0	0.5	12.03	44753.65	45340.12	17644.42	306148.43
706	800mg	4870.0	0.5	9.56	31719.22	31857.16	25112.10	346403.50

$AUC_{0-\infty}$, AUC, area under the concentration-time curve (AUC)extrapolated to infinity; C_{max}, maximum observed plasma concentration; CL/ F, apparent total clearance; T_{max}, time to maximum concentration; $t_{1/2}$, apparent terminal elimination half-life; V_z/F, apparent volume of distribution during the terminal elimination phase.
